# Diverse Virulent Pneumophages Infect *Streptococcus mitis*

**DOI:** 10.1371/journal.pone.0118807

**Published:** 2015-02-18

**Authors:** Siham Ouennane, Philippe Leprohon, Sylvain Moineau

**Affiliations:** 1 Département de Biochimie, Microbiologie et Bio-Informatique, Faculté des Sciences et de Génie, Groupe de Recherche en Écologie Buccale, Félix d’Hérelle Reference Center for Bacterial Viruses, Faculté de Médecine Dentaire, Université Laval, Québec City, Québec, Canada; 2 Département de Microbiologie, Infectiologie et Immunologie, Faculté de Médecine, Université Laval, Québec City, Québec, Canada; University of Helsinki, FINLAND

## Abstract

*Streptococcus mitis* has emerged as one of the leading causes of bacterial endocarditis and is related to *Streptococcus pneumoniae*. Antibiotic resistance has also increased among strains of *S*. *mitis* and *S*. *pneumoniae*. Phages are being reinvestigated as alternatives to antibiotics for managing infections. In this study, the two virulent phages Cp-1 (*Podoviridae*) and Dp-1 (*Siphoviridae*), previously isolated from *S*. *pneumoniae*, were found to also infect *S*. *mitis*. Microbiological assays showed that both pneumophages could not only replicate in *S*. *mitis* but also produced more visible plaques on this host. However, the burst size and phage adsorption data were lower in *S*. *mitis* as compared to *S*. *pneumoniae*. A comparison of the genomes of each phage grown on both hosts produced identical nucleotide sequences, confirming that the same phages infect both bacterial species. We also discovered that the genomic sequence of podophage Cp-1 of the Félix d’Hérelle collection is different than the previously reported sequence and thus renamed SOCP.

## Introduction

The *Mitis* group of streptococci is a member of the viridans group of streptococci [VGS], which includes several species that reside in the human oral cavity and the upper respiratory tract. In the *Mitis* group, *Streptococcus pneumoniae* and *Streptococcus mitis* are closely related species, making discrimination between them very difficult [1]. Although both bacteria are oral commensals they can cause a wide variety of human invasive diseases [2]. *S*. *pneumoniae* (pneumococcus) is the most common cause of community-acquired pneumonia worldwide and is also associated with a range of other diseases, including otitis media, meningitis, and septicemia [3]. *S*. *mitis* has traditionally been regarded as an innocuous commensal of the oropharynx, skin, and both the gastrointestinal and genitourinary tracts [4]. Some *S*. *mitis* strains have been shown to cause endocarditis and blood stream infections [5]. The transition from commensalism to pathogenesis is likely related to the acquisition of virulence genes.

*S*. *pneumoniae* and *S*. *mitis* have emerged as significant pathogens but differ in their expression of virulence factors. For example, phase variation is an important step in virulence and also an adaptive process that leads to the distribution of bacteria to other sites, causing disease [6]. The switching of phenotypes was noted in *S*. *pneumoniae* where phase variation changes the colony morphology from opaque to transparent without any change in the serotype. To our knowledge, this type of phase variation has not been observed for *S*. *mitis* [7], however, strains of *S*. *mitis* were recently shown to have a capsule, a well known virulence factor in *S*. *pneumoniae* [8]. In fact, genomic analysis of *S*. *mitis* revealed the presence of several virulence factors similar to those found in *S*. *pneumoniae*. However, it has not been confirmed whether these virulence factors are just implicated in adhesion and attachment of *S*. *mitis* or whether they are related to pathogenicity as in *S*. *pneumoniae* [5,9].

Strains nonsusceptible to the common antimicrobial drugs were detected among *S*. *pneumoniae* and *S*. *mitis*, especially to beta-lactams which have been widely used to treat such bacterial infections [10]. It was also reported for *S*. *pneumoniae* and other bacterial species that low concentrations of some antibiotics induces natural transformation and mutagenesis [11,12,13]. The ubiquitous nature of antibiotic resistance genes may also be due to horizontal gene transfer among strains of the naturally transformable *Mitis* group. Therefore, there is pressure to develop novel antibacterial alternatives.

Virulent phages may represent such an alternative strategy to combat antibiotic-resistant bacteria. Phages are recognized as the most abundant biological entities in the biosphere [14]. The study of phages of hemolytic streptococci began decades ago [15], and the vast majority of phages currently isolated from *S*. *mitis* and *S*. *pneumoniae* are temperate phages. In fact, both bacterial species contain many prophages in their genomes [16,17]. Prophage carriage was identified in clinical isolates of *S*. *pneumoniae* by hybridation and also via induction by mitomycin C [16,17]. At least seven phage-related gene clusters were detected in the genome of *S*. *mitis* B6 and two complete prophages (SM1 and phiB6) were isolated and sequenced [18,19]. Because temperate phages have the ability to transfer host DNA, which may encode virulence genes or toxins, into other bacterial strains [18,20], virulent phages are thought to be better suited for biocontrol purposes.

Very few lytic phages have been isolated for either of these two bacterial species. Despite the isolation of pneumophages, also called Omega phages [21], only two virulent pneumococcus phages are readily available today. Phage Dp-1 was the first virulent pneumophage isolated in 1975 [21,22] and belongs to the *Siphoviridae* family. Phage Cp-1 was isolated in 1981 [23] and is a member of the *Picovirinae* subfamily of the *Podoviridae* family, with a linear double-stranded DNA genome [24]. One virulent phage of *S*. *mitis*, vB_SmM_GEC-SmitisM_2, was isolated in 2012 from sewage water and belongs to the *Myoviridae* family [25].

Of interest, enzymes from virulent pneumococcal phages have also shown promise as antimicrobials [26,27,28,29,30,31,32]. At the end of their lytic cycle, virulent phages produce an enzyme (endolysin or lysine) that degrades the bacterial peptidoglycan to release new virions. A number of studies have demonstrated the potential of the lysin [Cpl-1] from phage Cp-1 to eradicate nasopharyngeal colonization and bacteremia [28], and to prevent otitis media [30]. The administration of Cpl-1 by inhalation [27] or by repetitive intraperitoneal injections even rescued mice from severe pneumococcal pneumonia [32].

In this study, we show that the virulent pneumophages Dp-1 and Cp-1 can infect *S*. *mitis*. Microbiological assays and genomic analyses were performed to compare phage behavior in both bacterial species.

## Materials and Methods

### Bacterial strains and culture conditions

The unencapsulated strain *S*. *pneumoniae* R6 (host of phage Cp-1), a derivative of *S*. *pneumoniae* strain R36A [host strain of phage Dp-1], was grown on TSA (Trypticase Soy Agar) containing 5% sheep’s blood at 37°C in the presence of 5% CO_2_. Single colonies were suspended in filtered BHI (Brain heart infusion) broth containing 0.5% yeast extract and incubated at 37°C in the presence of 5% CO_2_. *S*. *mitis* CCRI-15019 was grown at 30°C in BHI supplemented with 0.2 mM magnesium sulfate and 0.25 mM calcium chloride to prevent cell aggregation. The bacterial strains are stored at the Félix d’Hérelle Reference Center for Bacterial Viruses (www.phage.ulaval.ca).

### Bacterial species identification

The bacterial housekeeping genes *recA*, *recP*, *HexB*, and *xpt* were amplified by PCR using the primers listed in S1 Table [22]. The PCR products were sequenced by the Plateforme de Séquençage et de Génotypage des Génomes service at the CHUL/CHUQ Research Center using the ABI data 3730XL DNA analyzer. Both DNA strands of the amplicons were sequenced using the same primer pairs used for PCR amplification. Sequences were aligned and analysed using the bioinformatics tools (Clustal W2 and BioEdit).

### Phages

Pneumophages Cp-1 [23] and Dp-1 [22] were obtained from the Félix d’Hérelle Reference Center for Bacterial Viruses. For phage amplification, and to facilitate enumeration, we used liquid BHI+ medium, which consisted of BHI supplemented with 8 μM MnCl_2_, 0.25 mM CaCl_2_, 0.2 mM MgSO_4_, 50 Mm Tris-HCl pH 7.5, 50 ng/μl choline chloride, 0.4% glycine, and 100 μl/ml catalase. Amplifications of phage Cp-1 on *S*. *pneumoniae* and on *S*. *mitis* were performed with agitation, as follows: a single colony, isolated from overnight culture on BHI blood agar, was used for cultivation in BHI. After overnight growth of the host strains an aliquot of the culture was transferred into fresh BHI and incubated until it reached an optical density at 600 nm (OD_600nm_) of 0.06–0.08. The culture was diluted with an equal volume of BHI+, phages were added and incubated overnight at 30°C. Phage titers were obtained using a standard, double-layer agar assay method. To maximize plaque visualization, the bottom layer of BHI+ contained 1.5% agarose while the top agar contained 0.4% agarose.

### Microbiological assays

Phage lytic development was assessed using a one-step growth curve assay, in triplicate, as described elsewhere [33]. After overnight growth of the host strain in BHI, an aliquot of the culture was transferred into fresh BHI and incubated until it reached an OD_600nm_ of 0.06–0.08. The bacterial culture was diluted 1:3 in BHI+ and then infected at a multiplicity of infection of 0.05 at 30°C. Phages were allowed to adsorb to the host cells for 10 min. Unadsorbed phages were removed by centrifugation, the bacterial pellet was washed twice with BHI media and then we proceeded as described elsewhere [33]. The plates were incubated at 30°C overnight with 5% CO_2_, and the plaque forming units (PFU) were counted. The burst size was determined by calculating the ratio of the average phage titer after the exponential phase to the average titer before the infected cells began to release virions [33]. Phage adsorption tests were also carried out on *S*. *pneumoniae* R6 and *S*. *mitis* CCRI-15019, in triplicate, essentially as previously reported [34]. Modifications: BHI+ broth was used, CaCl_2_ was not added again at phage infection and the incubation was carried out at 30°C for 10 min.

### Electron microscopy

Phage preparations produced on *S*. *pneumoniae* and *S*. *mitis* were purified using a CsCl gradient, as previously described [35]. Phages were then centrifuged and 100 μl of the pellet were kept and washed with 1.5 ml of ammonium acetate (0.1 M, pH 7.5). This process was repeated twice and 100 μl from the final wash was retained for observation by transmission electron microscopy. Grid preparation and observation were performed as previously described [36]. Phages were observed at 80 kV using a JEOL 1230 transmission electron microscope.

### Phage DNA preparation and sequencing

Genomic DNAs of phages Cp-1 and Dp-1 amplified on *S*. *mitis* CCRI-15019 were isolated using a Lambda Maxi Kit (Qiagen). Phage Cp-1 DNA was also isolated after propagation on *S*. *pneumoniae* R6. Genome sequencing was performed on a 454 FLX instrument at the Plateforme d’Analyses Génomiques of the Université Laval (IBIS). The genomic sequences were completed by primer walking (primer sequences are listed in S2 Table) and by sequencing of PCR products. All mutations were also confirmed by primer walking.

### Phage genome analyses

Genomic sequences were analyzed using BioEdit 7.2.0 (http://www.mbio.ncsu.edu/bioedit/bioedit.html) and the Staden package [37,38] (http://staden.sourceforge.net/). Sequence alignments were performed using BioEdit and Clustal W2 software (http://www.ebi.ac.uk/Tools/msa/clustalw2/). Open reading frames [ORFs] were identified using GenMark (http://exon.gatech.edu/) and ORFinder (http://www.ncbi.nlm.nih.gov/projects/gorf/). Sequences were considered to be ORFs if they showed a putative ribosome binding site (RBS) at a reasonable distance from the starting codon (AUG, UUG, or GUG) and they consisted of at least 29 amino acids (aa). Function was attributed to an ORF by comparing the translated product to proteins available at the National Center for Biotechnology Information (BLASTp, http://blast.ncbi.nlm.nih.gov/Blast.cgi). The annotations were reinforced by searching for protein functional domains using the NCBI Conserved Domain Database (http://www.ncbi.nlm.nih.gov/Structure/cdd/wrpsb.cgi) and EMBL InterProScan (http://www.ebi.ac.uk/Tools/InterProScan/). The theoretical molecular masses [MM] and isoelectric points [pI] of each deduced phage protein were determined using the ProtParam software tool available on the bioinformatics resource portal ExPASy Web site (http://ca.expasy.org/tools/protparam.html). The genomes of phages Cp-1 and Dp-1 were searched for tRNAs using tRNAscan-SE [39] and BLASTn from NCBI. Bacterial codon usage for the host strains was obtained from the Kazusa DNA Research Institute database (http://www.kazusa.or.jp/codon/) while codon usage for the phages was determined using the DNA 2.0 web server (Menlo Park, CA); the frequency per thousand codons was then calculated.

The complete genome sequence of phage SOCP has been deposited in GenBank under accession number KJ617393.

## Results

### Speciation of bacterial hosts

The species identification of *S*. *mitis* from the closely related *S*. *pneumoniae* represents a challenge. For example, significant sequence conservation of the 16S rRNA gene sequence within the Mitis group limits the use of these sequences for species differentiation [40]. To confirm the bacterial strains used in this study, degenerate primers were used and the PCR products of four housekeeping genes, *recA*, *recP*, *hex B* and *xpt* (S1 Table) [40] were amplified and sequenced. Our results confirmed that the isolate CCRI-15019 is highly related to *S*. *mitis* B6 [18]. Our *S*. *pneumoniae* R6 and R36A strains were similarly confirmed.

### Morphological analysis of phages Dp-1 and SOCP

**Fig 1:**
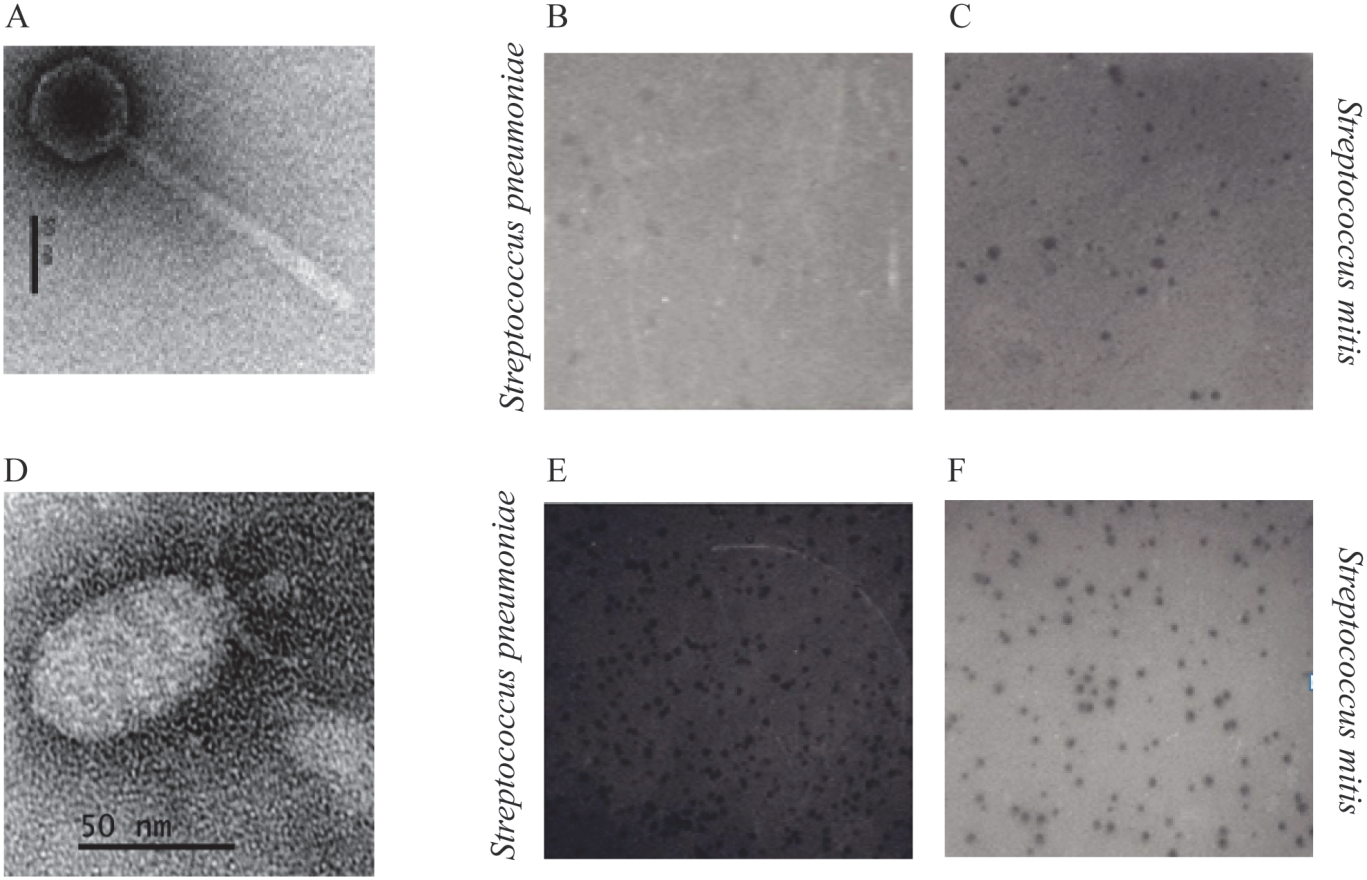
Electron microscopy of virulent phage Dp-1 (A) and phage SOCP (D). Scale bars correspond to 50 nm. Photographic images (B, C, E, F) show plaques of phages Dp-1 and SOCP propagation in media containing agarose. Plaques produced by phage Dp-1 on strains *S pneumoniae* R6 (B) and *S*. *mitis* CCRI-15019 (C). Plaques produced by phage SOPC on strains *S pneumoniae* R6 (E) and *S*. *mitis* CCRI-15019 (F).

As documented previously [40], phage Dp-1 belongs to the *Siphoviridae* family. It has an icosahedral capsid with an estimated diameter of 69.8 ± 1.2 nm (Fig. 1A) and a long non-contractile tail of 169.9 ± 3.6 nm in length and 19.6 ± 2 nm in width. Phage Cp-1, used in this study and stored at the d’Hérelle Center, was re-named SOCP due to genome variations (see below). Phage SOCP belongs to the *Podoviridae* family and has a hexagonal capsid of 65.8 ± 1.1 nm (top to bottom) and 42.1 ± 1.7 nm in width (Fig. 1D). This phage has a short non-contractile tail, 19.3 ± 1 nm in length and 7.5 ± 1.2 nm in width. Observations of phages Dp-1 and SOCP, amplified on both bacterial species, confirmed that the general morphology of these virions was not affected by growth in either hosts.

### Microbiological assays

One of the great difficulties of working with virulent pneumophages is the ability to visualize plaques consistently. We were able to observe plaques for both phages using BHI+ media (Figs. 1B & E). We also noticed that liquid cultures of *S*. *pneumoniae* grew better in BHI+ when started from colonies grown on blood agar. Finally, replacing agar by agarose in both top and bottom media led to an improvement in the plaque size and visibility.

The availability of an improved plaque assay allowed us to investigate the host range of phages Dp-1 and SOCP. Interestingly, we observed that they could both replicate on a *S*. *mitis* strain (CCRI-15019). Moreover, even larger phage plaques were obtained on *S*. *mitis*, compared to *S*. *pneumoniae* host cells grown under the same conditions (Figs. 1BC & EF). To further investigate the behavior of the two phages on both bacterial species, one-step growth curve assays were performed using *S*. *pneumoniae* R6 and *S*. *mitis* CCRI-15019. The latent period of phage Dp-1 was calculated to be 71 ± 4 min when amplified on *S*. *pneumoniae* and 59 ± 4 min on *S*. *mitis*. The burst sizes were 73 ± 6 plaque-forming units (PFU) per infected cell and 63 ± 6 PFU for *S*. *pneumoniae* and *S*. *mitis*, respectively. Similarly, the latent period of phage SOCP was estimated to be 78 ± 2 min on *S*. *pneumoniae* and 66 ± 5 min on *S*. *mitis*, while the burst size was 94 ± 4 PFU on its pneumococcal host and 53 ± 4 PFU on *S*. *mitis*. These data indicated a lower burst size for both phages on *S*. *mitis* cells but a shorter latent period.

Adsorption to the cell surface was also evaluated for the two phages on both strains. Under the conditions tested, the percentage of adsorption of phage Dp-1 on *S*. *pneumoniae* R6 was 95% ± 1.8% and was 79% ±1% on *S*. *mitis*. The adsorption of phage SOCP was 94% ± 0.6% on *S*. *pneumoniae* R6 and 86 ± 1.3% on *S*. *mitis* B6. Thus, both pneumophages could also readily adsorb to the *S*. *mitis* cell surface.

The efficacy of plaquing (EOP) of both phages on the two propagating hosts was determined. When phages Dp-1 and SOCP were amplified on their *S*. *pneumoniae* hosts they had EOPs of 10^–1^ and 10^–4^ on *S*. *mitis*, respectively. Similarly, when Dp-1 and SOPC were amplified on *S*. *mitis*, their EOPs on *S*. *pneumoniae* were 10^–1^ and 10^–3^, respectively. These data suggest the presence of host factors that modify the phage behavior.

### Genome analysis

To determine if replicating these phages on various hosts had an impact at the nucleotide level, we determined the complete genome sequence of both phages after amplification on *S*. *pneumoniae* or *S*. *mitis*. The double-stranded DNA (dsDNA) genome of phage Dp-1 is made of 56,506 bp. The analysis of the sequence found no nucleotide variation compared to the genomic sequence recently reported for Dp-1 [41]. Moreover, the sequence was identical when Dp-1 was amplified on both *S*. *pneumoniae* and *S*. *mitis* hosts, indicating that propagating this siphophage on two distinct streptococcal species did not lead to genomic changes.

Similarly, the genome of the podophage was also identical when propagated on either *S*. *pneumoniae* or *S*. *mitis*. However, analysis of the genomic sequence of the phage Cp-1 used in this study showed variations compared to the GenBank sequence no. NC_001825.1. As indicated above, we thus renamed our Cp-1-like phage SOCP due to these differences. The dsDNA genome of SOCP is 19,347 bp long, while the reported genome of phage Cp-1 is 19,343 bp. Comparative analyses revealed 31 variations in the genome of SOCP as compared to Cp-1 (S3 Table). These differences were confirmed through primer walking directly on the phage genome.

**Fig 2:**
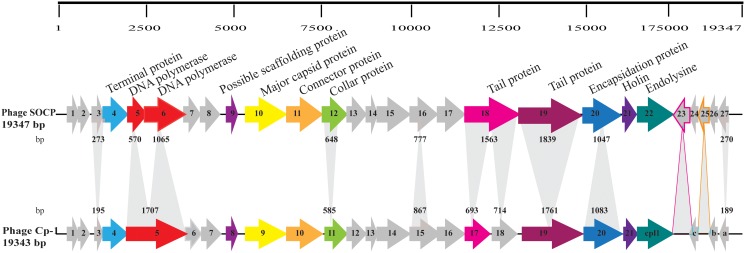
Genome alignment of phages SOCP and Cp-1. The scale above the phage genome SOCP is in base pairs. Each arrow represents a gene, and the numbering for SOCP refers to Table 1. Some putative functions of the deduced proteins are indicated above the arrows (see Table 1 for details). Grey arrows indicate that no putative function was attributed to the deduced protein. Arrows with the same color indicate the same general function and have at least 90% identity at the amino acid level. Heterologous regions are shaded gray, and the shaded numbers indicate the number of base pairs between the corresponding gene sequences. Shadows light with colored contour highlight ORFs not found in the genome of phage Cp-1.

The genome of phage SOCP has a GC content of 38.8%. It has 27 open reading frames, each preceded by a putative ribosome-binding site (RBS) (Table 1). Putative functions could be attributed to only 12 ORFs (44%). Interestingly, we found two additional ORFs in SOCP as compared to Cp-1, namely ORF23 and ORF25 (Fig. 2). Of interest were also the products of genes *orf5* and *orf6*, which likely code for DNA polymerase subunits [42,43], whereas only one (*orf5*) was found in Cp-1. Conversely in the case of *orf18*, one gene was found in SOCP whereas it was split into two genes (*orf17* and *orf18*) in Cp-1. The major capsid protein ORF10 was also affected by mutations, although its size remained the same (S3 Table). Other notable ORFs containing mutations included the tail protein ORF19, the collar protein ORF12, as well as ORF4, ORF9, ORF12, ORF16, ORF23, ORF25, and ORF27.

**Table 1:** Putative Open Reading Frames deduced from SOCP genome sequences and their predicted functions.

ORF	Start	End	Strand	Length [aa] ^a^	IP ^b^	Mw [kDa] ^c^	Putative RBS ^d^ and start codon	Putative function	Best hit with Blast ‘Locus tag’	ORF in Cp-1	# of identical aa/ size of the alignment [% aa identity]	Length [aa]	E-value	Accession number [GenBank] ^e^
**1**	376	642	+	88	4.4	10.4	**AAAGGAG**AAAGAAAACATG	Hypothetical protein	Cp-1, p01	1	88/88[100%]	88	3E-57	NP_044813.1
**2**	657	947	+	96	6.6	11.7	**AAAGGAG**ATAATAAAAATG	Hypothetical protein	Cp-1, p02	2	96/96[100%]	96	5E-61	NP_044814.1
**3**	1074	1346	+	90	4.7	10.5	**AAAGGAG**TAAAAGCACTTG	Hypothetical protein	Cp-1, p03	3	63/64[98%]	64	3E-36	NP_044815.1
**4**	1351	2043	+	230	10.1	26.7	**AAGG**G**G**TGTAATTAAATG	Terminal protein	Cp-1, p04	4	229/230[99%]	230	7E-165	NP_044816.1
**5**	2040	2609	+	189	4.6	22.1	**AAAGAAG**CGAGGGAAGAAGTG	DNA polymerase	Cp-1, p05	5	178/179[99%]	568	3E-123	NP_044817.1
**6**	2681	3745	+	354	6.8	40.8	**AAT**CTT**AGA**TGAAAAGGTG	DNA polymerase	Cp-1, p05	5	341/354[96%]	568	0	NP_044817.1
**7**	3702	4148	+	148	9.2	17.6	**AAAGG**G**G**GTACGCTGATTTATG	Hypothetical protein	Cp-1, p06	6	148/148[100%]	148	1E-100	NP_044818.1|
**8**	4141	4653	+	170	4.8	18.9	**AAA**C**GGAG**ATAAACAAAATG	Hypothetical protein	Cp-1, p07	7	170/170[100%]	170	5E-118	NP_044819.1
**9**	4875	5165	+	96	4.2	10.5	**AAAGGAG**AGGGCTATG	Scaffolding protein	Cp-1, p08	8	95/96[99%]	96	4E-59	NP_044820.1
**10**	5409	6506	+	365	5.4	41.7	**AAGAGG**GAGAAGAATAGAATG	Major head protein	Cp-1, p09	9	352/365[96%]	365	0	NP_044821.1
**11**	6563	7576	+	337	5.3	39.5	**AAAGG**G**G**ACTAAATG	Connector protein	Cp-1, p11	10	337/337[100%]	337	0	NP_044823.1
**12**	7563	8210	+	215	5.1	24.7	**AAAAGGAG**GGGACAATCATTG	Collar protein	Cp-1, p12	11	192/194[99%]	194	2E-137	NP_044824.1
**13**	8223	8807	+	194	8.6	22.8	**AAAGG**T**G**TATAGATG	Hypothetical protein	Cp-1, p13	12	194/194[100%]	194	5E-140	NP_044825.1
**14**	8804	9118	+	104	5.8	11.9	**AAAGAGG**ACATGAAAACCTATG	Hypothetical protein	Cp-1, p14	13	104/104[100%]	104	7E-67	NP_044826.1
**15**	9102	9989	+	295	4.9	32.9	**AAAAAGAGG**TAGAAACAAATG	Hypothetical protein	Cp-1, p15	14	295/295[100%]	295	0	NP_044827.1
**16**	10011	10787	+	258	5.0	29.4	**AAAGGA**TTTTAAAACATG	Hypothetical protein	Cp-1, p16	15	192/207[93%]	288	4E-132	NP_044828.1
**17**	10787	11548	+	253	6.0	28.4	**AGGAG**GTATCTAATG	Hypothetical protein	Cp-1, p18	16	253/253[100%]	253	0	NP_044830.1
**18**	11515	13077	+	520	5.7	59.0	**AAAG**TC**G**GGTCAATG	Tail protein	Cp-1, p19 / Cp-1, p20	17 18	210/224[94%]/ 236/237[99%]	230 /237	6e-149/ 2E-164	NP_044831.1 / NP_044832.1
**19**	13081	14919	+	612	4.8	67.5	**AAAGG**GTAAACAATG	Tail protein	Cp-1, p21	19	582/583[99%]	586	0	NP_044833.1
**20**	14993	16039	+	348	7.6	40.8	**AAA**T**GG**TACAATCCGCAGAAAATG	Encapsidation protein	Cp-1, p23	20	348/348[100%]	360	0	NP_044835.1
**21**	16029	16433	+	134	7.9	15.5	**AGG**TTATCAATCATG	Holin protein	Cp-1, p24	21	134/134[100%]	134	1E-89	NP_044836.1
**22**	16433	17452	+	339	4.6	39.2	**AAAGGAG**AAAAGAAATAATG	Lysozyme	Cp-1, p25	22	339/339[100%]	339	0	NP_044837.1
**23**	17480	17896	-	139	5.62	15.8	**AAAA**C**G**T**AGGG**GGTTAATACTATG	Hypothetical protein	SP058_00395		24/65[37%]	234	6E+00	YP_008239483.1
**24**	17901	18143	-	80	9.9	9.4	**AAA**TT**GAGG**TATTAAGAAAATG	Hypothetical protein	Cp-1, p26 [orfc]	c	80/80[100%]	80	5E-50	NP_044838.1
**25**	18148	18423	-	91	5.1	10.9	**AAGGGA**CGGTTACTAGATG	Hypothetical protein	AGR66263		14/44[32%]	410	8E-01	gb|AGR66263.1
**26**	18424	18693	-	89	7.7	10.8	ACAA**A**T**AGGAG**GGTAAACATG	Hypothetical protein	Cp-1, p27[orfb]	b	89/89[100%]	89	5E-58	NP_044839.1
**27**	18704	18973	-	89	5.0	10.2	**AAAGAGG**TATAACAAAATG	Hypothetical protein	Cp-1, p28 [orfa]	a	60/61[98%]	62	7E-35	NP_044840.1

^a^ Number of amino acids (aa),

^b^ IP, isoelectric point and

^c^ MM, molecular mass.

^d^ RBS, ribosomal binding site. Bases in bold correspond to nucleotides identical to the RBS consensus; lowercase indicates.

Three open reading frames were identified on the negative strand of the SOCP genome, specifically within three other ORFs (major capsid protein, tail protein and one hypothetical protein). These ORFs were not included in the annotation of the phage genome since no BLAST data support these proteins as valid protein products.

### Codon usage

No tRNAs were found in either of the two pneumophages. Still, the codon usage frequency was investigated for the phage genomes and compared with the codon usage of *S*. *pneumoniae* and *S*. *mitis* (S4 Table). The codon usage of pneumophages mostly corresponds to the codons most frequently used by these two bacteria, although the codon usage of the phages was closer to *S*. *pneumoniae*, suggesting that both phages are mode adapted to this bacterial species. In a few cases, some codons were over-represented in the phages as compared to the two bacterial hosts, likely to favor phage multiplication (S4 Table).

### Comparative genomics

**Fig 3:**
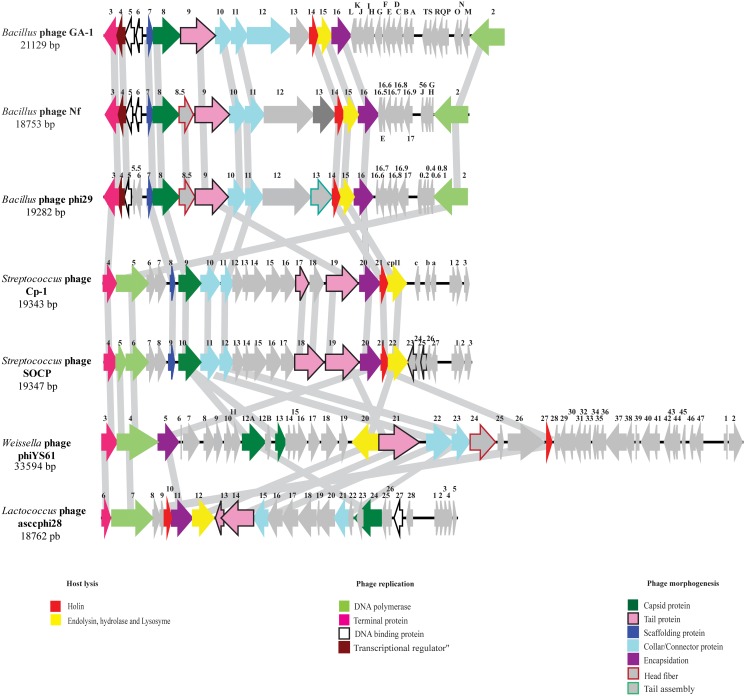
Comparison of the proteome of pneumophage SOCP/ Cp-1 with other related phages replicating through a protein priming DNA replication system. Phage genome sequences were downloaded from GenBank, aligned in BioEdit and each deduced protein was compared using BioEdit and protein BLAST. Arrows of the same color correspond to ORFs with the same general function.

Protein-primed DNA replication was reported for phage Cp-1 [44]. Few other phages of the *Podoviridae* family replicate using this mechanism of DNA replication [44,45,46] and, in general, initiation of replication arises at the 3’ nucleotide of the DNA [44]. The genomes of phages SOCP and Cp-1 were aligned with the genomes of five phages infecting other Gram-positive bacteria, namely *Bacillus subtilis* phage phi29, *Lactococcus lactis* phage asccphi28, *Weissella cibaria* phage phiYS6 as well as *Bacillus* sp. phages GA-1 and Nf (Fig. 3). The genome organization of the two pneumophages is somewhat different from the other phages due to alternate orientations of some genes, although a few annotated proteins are related (Fig. 3). The genes coding for the DNA polymerase and terminal proteins are on the positive strand in one group (phage Cp-1, SOCP, phiYS61 and asccphi28) and on the opposite strand for another group (phage GA-1, Nf and phi29). Moreover, the genes for structural proteins in the lactococcal phage asccphi28 are in the opposite direction, compared to the other podophages shown in Fig. 3. Overall, a low level of identity was found between phages Cp-1/SOCP and these podophages, which is likely due to their distinct ecological niches and host strains.

## Discussion

*S*. *mitis* is a species closely related to *S*. *pneumoniae* that colonizes the human oral cavity. Both species have emerged as multidrug-resistant pathogens due to their ability to acquire foreign DNA by natural competence. The genome of *S*. *mitis* has significant similarity to the genome of *S*. *pneumoniae* and shares a core of 900 genes [18]. Since the publication of the *S*. *mitis* B6 genome, studies have been carried out to understand this relationship and a number of studies support the hypothesis of evolution of *S*. *pneumoniae* from *S*. *mitis* by acquisition of virulence factors and numerous sugar-related transport systems [18,47]. Other studies have proposed that another possible concept is the evolution from a pathogenic bacterium, *S*. *pneumoniae*, to a commensal, *S*. *mitis*, through loss of virulence genes [18,40]. Virulence genes could also have been acquired through horizontal gene transfer between other related species [48]. Many virulence factors are cell surface proteins that play a role in the interaction with host cells [49]. Other pneumococcal cell surface components such as choline-containing teichoic acid, are components of the Dp-1 phage receptors [50].

Here, we report that two pneumophages can efficiently replicate on a *S*. *mitis* strain. These two virulent phages were initially isolated from throat swabs of patients with upper respiratory infections—the natural habitat of *S*. *mitis*. The ability of phage Dp-1 to infect *S*. *mitis* may be related to the choline-binding proteins found in *S*. *mitis* [50]. Homologues of pneumococcal surface choline-binding proteins are present in *S*. *mitis* but their role in this bacterium remains unknown [5]. The host autolysin system, involved in pneumophage virion release [51], is also found in both *S*. *pneumoniae* and *S*. *mitis* [52]. The adsorption of phages Dp-1 and SOCP was slightly higher on the *S*. *pneumoniae* host compared to *S*. *mitis* and this may be due to the availability of phage receptors on the cell surface or differences in receptor sequences. It was previously reported that phage Cp-1 adsorbed very poorly to the host cell surface [52]. Phage SOPC adsorbed very well to its host as well as the *S*. *mitis* strain. This could be due to the mutations observed in the genome of SOCP or the experimental conditions used in our study.

The burst size and latent period reported here are rather different from previous studies for both pneumophages [23,53]. Again, this could be due to the difference in experimental conditions and media used. For phage Dp-1, the host bacterial strain used by Lopez *et al*. [53] was *S*. *pneumoniae* R36A and in our study we used the *S*. *pneumoniae* R6, a derivative of R36A. Likewise for phage SOCP, its burst size was much higher than reported for Cp-1 [41]. It is not known if this difference is due to the experimental conditions or the genomic variations. Of note, it was previously reported that phage Cp-1 can infect some *Streptococcus oralis* strains [54]. SOCP can also infect a few *S*. *oralis* strains (data not shown).

In addition to the observations that pneumophages can replicate on *S*. *mitis*, the other most striking finding was the difference (0.16%) in the genome sequences between SOCP and Cp-1. The nature of such discrepancies remains unclear. Phage SOCP was retrieved from the Félix d’Hérelle Reference Center for Bacterial Viruses and was initially believed to be Cp-1. Either sequencing errors occurred in the original deposited sequence (GenBank no. Z47794) or a very efficient pneumophage was recovered. The latter could be due to phage host-adaptation, giving SOCP the ability to efficiently infect its hosts [55]. It has been reported that viral adaptation occurs through numerous mutations in the genome at low frequency that increases fitness of the phage [55,56]. It will thus be interesting to assess whether some of the nucleotide variations detected in SOCP are enabling such increased fitness.

This study on virulent phages provides another example of the relatedness of *S*. *pneumonia* and *S*. *mitis*. Both species also carry numerous temperate phages in their genomes that are probably involved in their evolution. Further study is needed to identify most of the hypothetical genes in phage SOCP as well as to increase our understanding of phage-host interactions [57], with a goal of eventually using virulent streptococcal phages in medical applications.

## Supporting Information

S1 Table Primer sequences of four housekeeping genes. F, forward; R, reverse.
(DOCX)

S2 Table Primers used in this study to confirm variations within the genome of phage SOCP. F, forward; R, reverse.
(DOCX)

S3 Table Differences between the genomes of phage SOCP and Cp-1. ^a^ relative to the genome of phage Cp-1.—absence of the nucleotide.
(DOCX)

S4 Table Codon usage of pneumophages Dp-1 and SOCP for selected amino acids compared to the hosts *S*. *pneumoniae* and *S*. *mitis*. The values represent the frequency of codon usage per thousand. The phage preferentially used codons for each amino acid are displayed in bold.
(DOCX)
