# Supplementary material for: Diverse Virulent Pneumophages Infect Streptococcus mitis
Source: PLoS One. 2015 Feb 18;10(2):e0118807. doi: 10.1371/journal.pone.0118807 (PMC4334900; doi:10.1371/journal.pone.0118807)
Supplement: S1 Table — F, forward; R, reverse. (DOCX) [file pone.0118807.s001.docx]

**S1 Table. Primer sequences of four housekeeping genes**

| Locus tag | Gene | Primer sequence 5’-3’ | Reference |
| --- | --- | --- | --- |
| *rec A* | Recombinase | F: GCCTT**Y**ATCGATGC**B**GA**R**CA  R: GTTTCCGG**R**TTDCC**R**AACAT | ([57](#_ENREF_57)) |
| *rec P* | Transketolase | F: ACCGCGACCGCTTTATTCTTTC  R: ATGCTGACTACGCGGGATTTTTC | ([54](#_ENREF_54)) |
| *hex B* | DNA mismatch repair | F: CCATTGACGCGGGCTCTA  R: CCTGAATACGTCGGAACATCTTT | ([54](#_ENREF_54)) |
| *xpt* | Xanthine phosphoribosyltransferase | F: GAAATTATTAGAAGA**R**CGCATC  R: TTAGAGATCTGCCTCC**W**TA**R**AA | ([54](#_ENREF_54)) |

F, forward; R, reverse.
