# Supplementary material for: Diverse Virulent Pneumophages Infect Streptococcus mitis
Source: PLoS One. 2015 Feb 18;10(2):e0118807. doi: 10.1371/journal.pone.0118807 (PMC4334900; doi:10.1371/journal.pone.0118807)
Supplement: S2 Table — F, forward; R, reverse. (DOCX) [file pone.0118807.s002.docx]

**S2 Table. Primers used in this study to confirm variations within the genome of phage SOCP.**

| **Primer pair name** | **Primer sequence (5’-3’)** | **PCR size (bp)** |
| --- | --- | --- |
| p1 | F : CGCTGATTTTAAACAATAATGTGAATATTTTG  R : TTCAAGTTTAATCTCATTTCCTAGGTCTAC | 1442 |
| p2 | F : CGTACTCAAGTATTTGGCAATCATG  R : GAGATTATGAAAGAATATGATAAATTCATCTTCTCC | 725 |
| p3 | F : GATTAAGCCAGAATGGATTGACTACA  R : CTCTATATACAGTTTAGAGCGTAAATATTTTG | 1054 |
| p4 | F : CCTTGAAAAGCCTTACAGGTTTATC  R : GAAAATTCTGCTGAGATAACCAGATATTG | 1425 |
| p5 | F : CGATTGCAACCTTTTTAAAATCCAGATATAG  R : GGTTTCTTTTAGTCCTGTGATGTTCA | 1094 |
| p6 | F : CTTTGCTTATCCGTAGATACATGGAG  R : CTATCTGTCCTTGTCCTATCCATTG | 1280 |
| p7 | F : GACGTTCTCTATATCAATGAAGATGCTAC  R : CCACTGTCCTATCCCAATACCTATC | 960 |
| p8 | F : GATAGGACAGTGGACGGGAC  R : CTAACTGTCTATCTCCTGTGTTACC | 814 |
| p9 | F : GCTATGACGTTATAGAACAAAATTATGC  R : GCTTCTGCGTCATTCAAAATAGC | 663 |
| p10 | F : GTCTCCCTAGCTCAAAAAGAAATCA  R : CGAGATACCTCTTTTTACTGGTAACG | 812 |
| p11 | F : GCTAGTAGGATTTTCCTACTAGCTG  R : GTTTCCAGACGATAAACTGGAAAC | 1167 |

F, forward; R, reverse.
