# Supplementary material for: Diverse Virulent Pneumophages Infect Streptococcus mitis
Source: PLoS One. 2015 Feb 18;10(2):e0118807. doi: 10.1371/journal.pone.0118807 (PMC4334900; doi:10.1371/journal.pone.0118807)
Supplement: S3 Table — a relative to the genome of phage Cp-1.—absence of the nucleotide. (DOCX) [file pone.0118807.s003.docx]

**S3 Table. Differences between the genomes of phage SOCP and Cp-1.**

| **Phage mutation** | | **Phage Cp-1** | | **Amino acid**  **variation** | |
| --- | --- | --- | --- | --- | --- |
| **SOCP** | **Cp-1** | **Position^a^** | **Putative function^a^** | **SOCP** | **CP-1** |
| G | C | 1870 | Terminal protein | Ala | Arg |
| C | G | 1871 | Terminal protein |  |  |
| - | T | 2579 | DNA polymerase | Leu | Phe |
| A | - | Between 2701-2102 | DNA polymerase | Ala | His |
| - | G | 2747 | DNA polymerase | Gly | Ala |
| A | G | 3116 | DNA polymerase | Thr | His |
| G | A | 3118 | DNA polymerase | Gly | Asp |
| T | C | 5036 | Scaffolding protein | Leu | Pro |
| - | T | 5197 | Non-coding region |  | |
| C | - | Between 5338-5339 | Non-coding region |  |  |
| A | - | Between 5959-5960 | Major capsid protein | Lys | Arg |
| - | C | 6003 | Major capsid protein | Asp | Thr |
| G | C | 8137 | Collar protein | Ala | Arg |
| C | G | 8138 | Collar protein |  |  |
| G | - | Between 10578-10579 | Hypothetical protein | Ala | Leu |
| A | - | Between 12206-12207 | Tail protein | Arg | Ser |
| G | - | Between 13027-13028 | Tail protein | Gln | His |
| G | - | Between 13027-13028 | Tail protein | Ala |  |
| G | - | Between 13163-13164 | Tail protein | Gly | Arg |
| A | T | 13669 | Tail protein | Glu | Val |
| C | G | 14955 | Encapsidation protein | Ala | Glu |
| G | - | Between 17703-17704 | Non-coding region |  | |
| G | - | Between 17799-17800 | Non-coding region |  |  |
| - | C | 18220 | Non-coding region |  |  |
| - | G | 18261 | Non-coding region |  |  |
| T | - | Between 18266-18267 | Non-coding region |  |  |
| G | - | Between 18266-18267 | Non-coding region |  |  |
| - | G | 18275 | Non-coding region |  |  |
| A | T | 18747 | Non-coding region |  |  |
| A | G | 18748 | Non-coding region |  |  |
| - | C | 18787 | Hypothetical protein | Asp | Glu |

^a^ relative to the genome of phage Cp-1.

- absence of the nucleotide.
