# Supplementary material for: Diverse Virulent Pneumophages Infect Streptococcus mitis
Source: PLoS One. 2015 Feb 18;10(2):e0118807. doi: 10.1371/journal.pone.0118807 (PMC4334900; doi:10.1371/journal.pone.0118807)
Supplement: S4 Table — The values represent the frequency of codon usage per thousand. The phage preferentially used codons for each amino acid are displayed in bold. (DOCX) [file pone.0118807.s004.docx]

**S4 Table. Codon usage of pneumophages Dp-1 and SOCP for selected amino acids compared to the hosts *S. pneumoniae* and *S. mitis.***

|  |  | **Phage** | | **Bacteria** | |
| --- | --- | --- | --- | --- | --- |
| **Amino acid** | **Codon** | **Dp-1** | **SOCP** | ***S. pneumoniae*** | ***S. mitis*** |
| Ala | GCG | 8.4 | 7.5 | **8** | 3.3 |
|  | GCT | 28.5 | 21.4 | 30.4 | **27.4** |
|  | GCC | 8.4 | 11.4 | 15.8 | **7.7** |
| Arg | AGG | 5.0 | 3.0 | 2.0 | **3.3** |
|  | AGA | 10.6 | 11.4 | 7.0 | **10.9** |
|  | CGG | 1.8 | 3.8 | **1.9** | 0.0 |
| Asn | AAC | 20.8 | 38.3 | 14.1 | **20.8** |
| Asp | GAC | 36.8 | 36.6 | 17.8 | 28.4 |
| Cys | TGC | 3.4 | 3.2 | **1.7** | 0.0 |
| Gln | CAA | 30.1 | 24.2 | **26.7** | 15.3 |
| Glu | GAA | 58.3 | 49.5 | **51.1** | 47.0 |
| Gly | GGC | 10.8 | 11.7 | **9.1** | 23.0 |
|  | GGG | 28.0 | 13.9 | 8.5 | 8.8 |
| Ile | ATC | 17.7 | 24.7 | **25.1** | 15.3 |
|  | ATT | 41.7 | 30.9 | **39.5** | 33.9 |
| Leu | TTA | 12.8 | 21.1 | **20** | 32.8 |
|  | CTG | 11.6 | 8.1 | **9** | 3.3 |
|  | CTC | 8.1 | 4.7 | **12.4** | 0.0 |
| Lys | AAA | 44.0 | 50.1 | **43.2** | 47.0 |
|  | AAG | 28.4 | 28.1 | 24.5 | **30.6** |
| Phe | TTC | 22.0 | 14.4 | **13.7** | 17.5 |
| Pro | CCC | 1.6 | 3.5 | 2.9 | **1.1** |
| Ser | AGC | 9.7 | 17.4 | **8.2** | 5.5 |
|  | TCA | 17.1 | 17.4 | 15.9 | **17.5** |
|  | TCC | 4.2 | 3.3 | 5.1 | **4.4** |
|  | TCG | 6.9 | 1.3 | 3.8 | **1.1** |
| Thr | ACA | 14.8 | 22.7 | 18.5 | **23.0** |
|  | ACC | 8.4 | 10.4 | **12** | 4.4 |
| Val | GTA | 15.4 | 18.5 | **14.4** | 23.0 |
|  | GTC | 14.2 | 8.0 | **15.1** | **8.8** |
|  | GTG | 8.1 | 10.4 | 12.2 | **8.8** |
|  | GTT | 27.4 | 18.0 | **27.4** | 25.2 |
| Tyr | TAC | 24.3 | 18.2 | 13.2 | **25.2** |
| End | TAA | 2.0 | 4.2 | **2.1** | 3.3 |
|  | TAG | 1.2 | 0.8 | **0.8** | 0.0 |

The values represent the frequency of codon usage per thousand.

The phage preferentially used codons for each amino acid are displayed in bold.
